# Occupancy State Prediction by Recurrent Neural Network (LSTM): Multi-Room Context

**DOI:** 10.3390/s23239603

**Published:** 2023-12-04

**Authors:** Mahamadou Klanan Diarra, Amine Maniar, Jean-Baptiste Masson, Bruno Marhic, Laurent Delahoche

**Affiliations:** Laboratory of Innovative Technologies (LTI UR 3899), Picardy Jules Verne University, 80000 Amiens, Francejean-baptiste.masson@u-picardie.fr (J.-B.M.); bruno.marhic@u-picardie.fr (B.M.); laurent.delahoche@u-picardie.fr (L.D.)

**Keywords:** machine learning, neural network, artificial neural networks, long short-term memory, occupant behavior, building energy consumption, habits of occupants

## Abstract

The energy consumption of a building is significantly influenced by the habits of its occupants. These habits not only pertain to occupancy states, such as presence or absence, but also extend to more detailed aspects of occupant behavior. To accurately capture this information, it is essential to use tools that can monitor occupant habits without altering them. Invasive methods such as body sensors or cameras could potentially disrupt the natural habits of the occupants. In our study, we primarily focus on occupancy states as a representation of occupant habits. We have created a model based on artificial neural networks (ANNs) to ascertain the occupancy state of a building using environmental data such as CO_2_ concentration and noise level. These data are collected through non-intrusive sensors. Our approach involves rule-based a priori labeling and the use of a long short-term memory (LSTM) network for predictive purposes. The model is designed to predict four distinct states in a residential building. Although we lack data on actual occupancy states, the model has shown promising results with an overall prediction accuracy ranging between 78% and 92%.

## 1. Introduction

At the beginning of the 20th century, the mass electrification of buildings led to a boom in household electrical technologies [1]. Nowadays almost all buildings are equipped with heating, hot water, and ventilation systems. This equipment represents the overwhelming majority of building energy consumption (77% in 2020 https://www.ceren.fr/ (accessed on 1 July 2022)). Given that buildings are generally occupied by several people who do not all have the same occupation habits, the effective use of this equipment by the occupants is, therefore, complicated in practice. It is, therefore, clear that the building sector (residential, tertiary) is an important breeding ground for reducing energy consumption. The problem of optimizing the energy consumption of buildings through the efficient use of the heating, domestic hot water, and ventilation systems was posed to the researchers. Many studies have sought to assess the impact of occupancy and/or occupants on the energy consumption of buildings. Indeed, Bing Dong et al. [2] showed that although building insulation and the number of occupants have an influence on energy consumption, it is the habits of the occupants that have the greatest correlation with consumption. To achieve this result, the authors looked at five types of housing with different insulation envelopes and different numbers of occupants. At the level of each building, motion sensors (PIR) have been installed as well as four power-monitoring systems to record consumption data. Kaiyu Sun and Tianzhen Hong [3] identified three occupant styles (austere, wasteful, and normal) and showed that the occupant style has a significant impact on energy consumption. The authors also showed that in the context of an occupant-independent energy consumption management system, energy consumption is weakly influenced by occupant style. Zhiyuan He et al. [4], like [3], sought to quantify the potential energy savings obtained by improving the behavior of the occupants. However, they used real survey data from Singapore. They considered four occupant styles (normal, wasteful, moderate, and austere) and incorporated occupancy models using Markov chains developed by Yixing Chen et al. [5]. When compared to the normal style, their work shows a 13.4% increase in consumption of the wasteful style, a 9.5% reduction in the moderate style, and a 21% reduction in the austere style. W. Zhang et al. [6] conducted a survey on the energy usage of 112 families in high-rise buildings and found that energy consumption and thermal satisfaction vary widely between occupants and that occupant behavior matters more than the quality and quantity of the equipment used for lowering energy usage. MS. Aliero et al. [7] showed that different control strategies must be used between commercial and residential buildings to account for occupant responses and unexpected variations in occupancy and weather conditions. Ashouri et al. [8] proposed a recommendation system that provides occupants with potential energy savings achievable based on past energy consumption patterns obtained with data-mining techniques (clustering, association rule, artificial neural networks). An efficient HVAC system is also important for occupants’ health; González-Lezcano [9] emphasized the need to maintain optimal indoor air quality to promote the well-being of inhabitants.

The correlation between the habits of people occupying a building and the energy consumption of the building being established, several tools have been developed to model these habits. J. Page et al. [10] used an inhomogeneous Markov chain to model the transitions between presence (1) and absence (0). The CDF inversion method is used to generate the occupancy profile. Shide Salim et al. [11] use an inhomogeneous Markov chain to predict transitions from one area to another in a workplace. Data were collected using a real-time locating system (RTLS). Transition probabilities are a function of the occupant, weather, and day of the week. Zhaoxuan Li et al. [12] also used Markovian modeling on the occupancy profile of a residential building. The transition matrices are estimated by maximum likelihood and the procedure is optimized using the Pearson divergence test to determine the best training window. The authors compare their method to different models (SVM, ANN, probability sampling) over different prediction horizons (15, 30 min, and 24 h). Their model shows better performance over the 15 and 30 min horizons and comparable performance for the 24 h horizon. Kabbaj, O.A. et al. [13] in their paper used hidden Markov chains to predict occupancy state from synthetic occupancy data. In practice, it is common to have missing data for several reasons including hardware and/or network problems which can lead to corruption or absence of data. The authors of this paper have developed a model adapted to this type of situation with interesting results on simulated data. Ardeshir Mahdavi et al. [14] use an empirical method based on the calculation of occupation frequencies for a given time interval. By thresholding, they distinguish the significant proportions. They exploit the occupancy status of an office obtained through a motion sensor. Their method shows performances comparable to those of Reinhart [15] and Page et al. [10]. Mohammad Saiedur Rahaman et al. [16] exploit the data generated by the employees of a shopping center. Each employee wears a low-energy Bluetooth beacon that emits a unique ID; four Bluetooth gateways scattered around the mall collect the ID (unique identifier) of nearby beacons, the detection interval and the variations of the indicator received signal strength. The information allows them to locate each employee carrying a beacon, in time and space (states). The authors compare different machine learning algorithms to determine the positions of employees from the intensity of the signals received (DT, RF, SVM, MLP, KNN) and show that the random forest performs better than the others. Jesica E.M. et al. [17] used LSTM networks combined with different classification algorithms (SVM, RF, MLP, KNN) to predict the number of occupants at three offices. Environmental data (CO_2_, temperature, etc.), the number of occupants, and the consumption of certain appliances were collected. Their strategy was to predict environmental variables via LSTM networks (one for each office) and rank the predictions. Their strategy offers good results and the random forest shows better performance than other classification algorithms. In papers [18,19], LSTM networks are also used to predict the occupancy state. Hamza Elkhoukhi et al. [20] use an LSTM network to predict CO_2_ concentration and merge this prediction with ventilation rate, normal CO_2_ concentration in the air, and the rate of generated CO_2_/person through a steady-state model to determine the number of occupants. Their model manages to predict the number of occupants with 70% accuracy. Marina Dorokhova et al. [21] in their paper use a k-means to estimate the occupancy state and an LSTM network trained with these states for the following ones. Their model predicts occupancy status (presence/absence) with over 97% accuracy. In papers [22,23,24,25], feedforward hidden layer neural networks called ELM (extreme learning machine) networks are used. For ELM networks, the weights entering the neurons of the hidden layer are generated randomly and are not learned, only the weights linked to the output layer are learned [26]. ELM networks show quite good performance in predicting the occupancy state. Having the right room occupancy profile is crucial for effective HVAC system control. Indeed, knowing the occupancy schedules can make it possible to establish a heating and ventilation schedule, and knowing the number of occupants can allow more effective control of this equipment. Yukun Yuan et al. [27] seek to minimize the power of the system that is being penalized by the comfort of the occupants. Finally, Seungwoo Lee et al. [28], after predicting the times of arrival, determine the preheating or ventilation time necessary for comfort in the room.

As we have seen, the prediction of the state of occupation by neural networks can be done according to two strategies. The first is to predict environmental variables and then infer the occupancy status from these predictions. The second strategy is to directly predict the state from observed data. In this work, we subscribe to the second strategy and propose a model for predicting the occupancy state of a building based on a priori labeling and the use of an LSTM network. We use an architecture that makes it possible to link the data of the different rooms of a building to provide a prediction of the occupancy state of all the rooms without restricting ourselves to only two states (presence/absence). The architecture we use (provided by tensorflow) has the advantage of making it possible to adjust a single network for all the rooms of a building, thus avoiding the difficult task of building and adjusting different architecture for each room.

## 2. Materials and Methods

### 2.1. Data Collection

**Figure 1:**
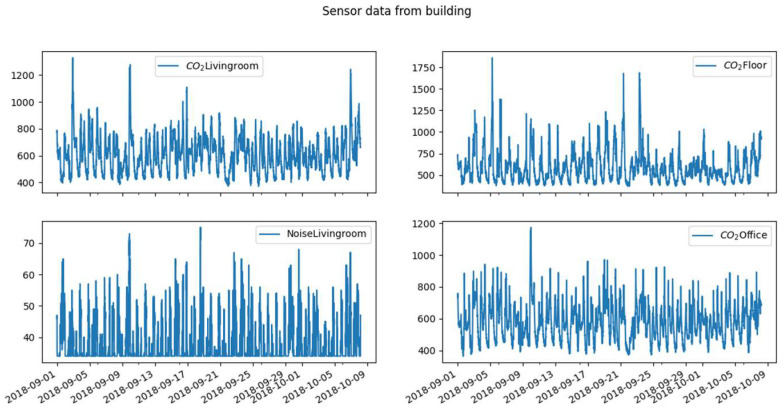
Sensor data graph.

In this work, we use unlabeled environmental data from a residential building in Amiens in the region of Picardie (northern France). The raw data were obtained through sensors (Netatmo equipment from Boulogne-Billancourt in France) placed in 3 rooms (living room, bedroom on the floor, and office) (Figure 1) of the building. The data, ranging from 1 September 2018 at 0:00 to 8 October 2018 at 4:40 are recorded at 5 min intervals. In each room, we have the evolution of the CO_2_ concentration in ppm, the living room is equipped in addition to the CO_2_ sensor with the noise sensor in dB. In Table 1 below, we can observe statistics on our variables in the different rooms. We notice that the medians and averages of CO_2_ are relatively close in the living room and the office which generally suggests a more or less symmetrical distribution. The difference between these two indicators (median and mean) is greater upstairs. We also notice that the maximum concentration of CO_2_ upstairs is higher than in the other two rooms, which may suggest either greater occupancy or less ventilation in this room. For the noise variable in the living room, we notice a significant difference between the median and the mean given the scale of values. We can also observe a very close proximity between the minimum and the median, which indicates that 50% of the noise levels are very close to the minimum. In other words, half the time, the noise level in the living room is close to minimum noise.

### 2.2. Methodology

**Figure 2:**
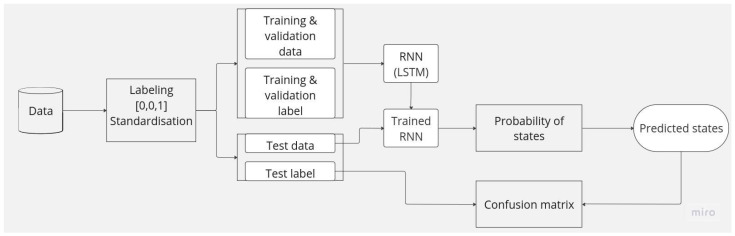
Diagrams of the methodology implemented in this work.

In this section, we describe the strategy implemented to answer our problem. As a reminder, we seek to anticipate the occupancy states of our various rooms based on data from environmental sensors. Occupancy states are defined in the section below. Figure 2 describes our methodology, which first consists of labeling a history of sensor data on the basis of binary rules. In the section below, we describe the adopted labeling rules. We then build the LSTM networks responsible for learning to predict the probabilities of the different occupancy states of one or more rooms.

#### 2.2.1. Preprocessing

The raw data we have are very noisy with temporal irregularities and missing data. To remedy this situation, we performed kernel smoothing of the data (Equation (1)). At the end of this procedure, the result is smooth data without temporal irregularities with regular resampling of data at 10 min intervals.

(1) $$x_{T}=\sum_{T-\frac{h}{2}}^{T+\frac{h}{2}}K\left(\frac{T-T_{i}}{h}\right)x_{T_{i}}$$

T: smoothing instant;

h: smoothing window;

K: core;

x: sensor data (CO_2_ or noise).

As we mentioned above (Section 2.1), we do not have the real occupancy states of the rooms. In this section, we describe on what basis we associated states with our data and how we chose the number of states.

A person continuously generates CO_2_ through exhalation, so in an enclosed space with no other source of CO_2_, an increase in the concentration of the latter necessarily indicates a presence. The same goes for noise, but to a lesser extent. Indeed, unless the room has sound insulation, the sensor can detect ambient noise, and the occupant can even be inactive and, therefore, not generating noise. Also note that there is an average concentration of CO_2_ in the atmosphere which was 412 ppm in 2020 according to the International Energy Agency. However, this average varies from region to region. In light of this information, we can draw the following conclusions:

- CO_2_ increasing and above a certain threshold indicates a presence;
- CO_2_ decreasing and above a certain threshold indicates an absence;
- Stable CO_2_ around the chosen threshold indicates a long absence;
- Adding noise allows us to register a fourth state: CO_2_ increasing and above the chosen threshold and no noise indicates an inactive presence.

To choose a CO_2_ threshold we use the work of Irvan B. et al. [29] in which the authors average the minimum CO_2_ concentrations below which a room is considered unoccupied. We carried out the same approach by taking the average of the daily minimums. We get an average of 445 ppm as the CO_2_ threshold. We then determined the different phases of CO_2_ increases. For this, we used the derivative of CO_2_ which is the appropriate characteristic for this. We use this information to associate occupancy states with our data via the rules set out below:

- State 0 (Prolonged absence): the CO_2_ derivative is in a tight range around the origin and the CO_2_ concentration is less than 445 ppm;
- State 1 (Presence): the CO_2_ derivative does not satisfy the conditions of the state (Prolonged absence) and is positive;
- State 2 (Absence): the CO_2_ derivative does not satisfy the conditions of the state (Prolonged absence) and is negative;
- State 3 (Presence without noise): the CO_2_ derivative does not satisfy the conditions of state 1 (Presence) and the noise level is low.

All labeling thresholds are given in the Section 3.2. After labeling, we put our data in the same scale of values by a mini-max normalization (Equation (2)). The CO_2_ concentration and noise were scaled between [0, 1]. This operation aims to increase the learning performance of the LSTM network. Indeed, this operation reduces the scale effect of the different variables and allows a faster convergence of the network.

(2) $$x_{\mathrm{stand}}=\frac{x-\min\left(x\right)}{\max\left(x\right)-\min\left(x\right)}$$

#### 2.2.2. Long Short-Term Memory (LSTM)

**Figure 3:**
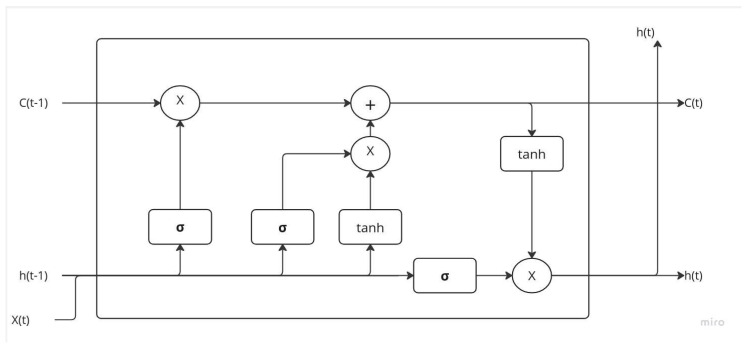
LSTM cell architecture.

This is the most popular RNN architecture, introduced in 1997 by Sepp Hochreiter and Juergen Schmidhuber [30] to overcome the problems of vanishing gradient as well as long-term dependence. For example, the value we are trying to predict at a time t could depend on a previous state in the distant past, while a classic RNN can only connect short-term dependencies. The idea is to split the input signal into two; a part that symbolizes the important information in the short term, called the hidden state which is in principle similar to the output of a classic RNN, as well as a part that symbolizes the important information on the long term, called the cell state. Figure 3 illustrates how LSTMs work overall. The information passes through different gates (portals) which control the flow of information inside the LSTM cell. They are called this because they act as filters that limit the information that can pass to the next cell. These gates allow you to:

- Detect relevant information from the past, stored in the cell state;
- Choose the information that will be relevant in the long term from the current input to update the cell state;
- Extract from the new cell state the relevant information in the short term to generate the hidden state.

#### 2.2.3. Experimental Parameters

For this work, we have one month and 8 days of smoothed and resampled data with 10 min intervals from 1 September 2018 to 8 October 2018, which we have broken down as follows:

- For the training data, we consider the period from 1 September 2018 at 0:00 to 23 September 2018 at 07:30, i.e., 22 days and 7:30.
- For the validation data, they range from 23 September 2018 at 7:40 to 30 September 2018 at 18:00, i.e., 7 days and 10:20.
- Finally, the test data range from 30 September 2018 at 6:10 to 8 October 2018 at 4:40.

The objective of this work, as indicated above, is to predict the occupancy status of the building from the observed sensor data. We use one hour of observed sensor data to predict the occupancy status over a one-hour horizon. Formally, let us say $x_{t}{,x}_{t+1},\ldots {,x}_{t+1H}$ one hour of observed sensor data, then we are looking for a function F:

(3) $$F\left(x_{t}{,x}_{t+1},\ldots {,x}_{t+1H}\right)=\left[P_{t+2H}\left({\mathrm{state}}_{1}\right){,P}_{t+2H}\left({\mathrm{state}}_{2}\right),\ldots {,P}_{t+2H}\left({\mathrm{state}}_{n}\right)\right]$$

(4) $${\mathrm{predictstate}}_{t+2H}=\mathrm{argmax}\left\{P_{t+2H}\left({\mathrm{state}}_{1}\right){,P}_{t+2H}\left({\mathrm{state}}_{2}\right),\ldots {,P}_{t+2H}\left({\mathrm{state}}_{n}\right)\right\}$$

**Figure 4:**
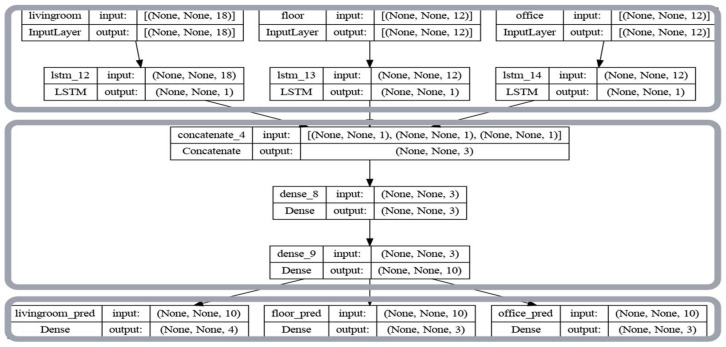
Architecture of the LSTM network we use.

In other words, we are looking for a function that from 1 h of data will return the probabilities of occupancy states at a specific time in the future; in this case on the expression above (Equation (3)) time in the future. The predicted occupancy state will, therefore, be the state with the greatest probability (Equation (4)). For multi-class problems (states), the most suitable activation function at the output of neural networks is the softmax which returns the probabilities of each state at the output. This is what we use in this work. We also want to take into account the interactions between the different parts of the building by adopting an appropriate architecture (Figure 4).

This architecture can be broken down into three blocks. A first parallel block consisting of the input layers and the LSTM cells. Each input layer takes data from a different room in the form of tables of 18, 12, and 12 columns for the living room, upstairs, and office, respectively. For the living room, 18 represents 1 h of observation, i.e., 6 time steps for the CO_2_ variable, the time derivative of CO_2_, and noise. The same applies to the other two rooms except that there is no noise sensor in these rooms. The data from each room are then fed to each lstm cell to extract a single feature for each one. A second sequential block made up of dense layers aggregates the outputs of the three lstm cells to produce a probability distribution for the states in each room (4 for the living room, 3 for the floor, and 3 for the office). The last block consists of parallel outputs, one for the probability distribution of the states in each room.

This architecture was used to extract features from three different rooms to feed the lstm network with more data without requiring an extended history of data, as well as to improve the model’s ability to generalize to different sets of inputs.

#### 2.2.4. Validation

To evaluate the performance of our occupancy state prediction strategy, we use criteria commonly used in classification: precision, recall, and f1-score. To better understand these criteria, let us stay on our problem. If our model predicts a presence, the precision gives us an indicator of confidence in this prediction. As for the recall, knowing the real state of occupation to come, the recall gives us an indicator of the capacity of our model to effectively predict the good state. These two criteria are expressed as follows:

(5) $${\mathrm{precision}}_{\mathrm{classi}}=\frac{\mathrm{numberofstatescorrectlyassociatedwithclassi}}{\mathrm{numberofstatesassociatedwithclassi}}$$

(6) $${\mathrm{recall}}_{\mathrm{classi}}=\frac{\mathrm{numberofstatescorrectlyassociatedwithclassi}}{\mathrm{numberofstatesbelongingclassi}}$$

In multi-class classification, model precision and recall are obtained by arithmetically averaging expressions (Equations (5) and (6)), respectively, as follows:

(7) $$\mathrm{precision}=\frac{1}{\mathrm{classnumber}}\sum_{i}{\mathrm{precision}}_{\mathrm{classi}}$$

(8) $$\mathrm{recall}=\frac{1}{\mathrm{classnumber}}\sum_{i}{\mathrm{recall}}_{\mathrm{classi}}$$

The f1-score is the geometric mean of expressions (7) and (8) above:

(9) $$f1-\mathrm{score}=2\frac{\mathrm{precision}*\mathrm{recall}}{\mathrm{precision}+\mathrm{recall}}$$

**Figure 5:**
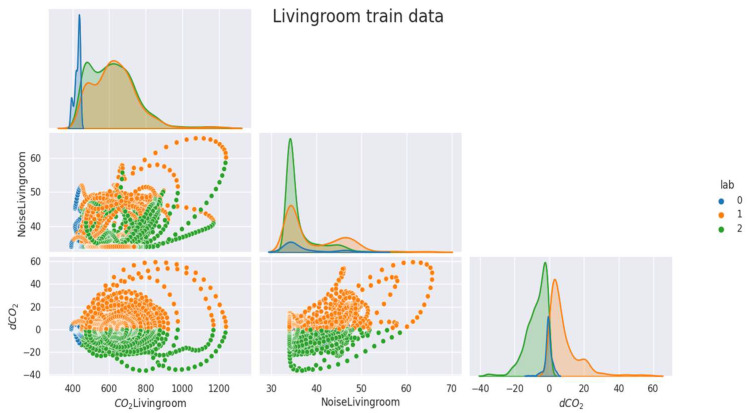
Distribution of data by class for the living room’s train data.

**Figure 6:**
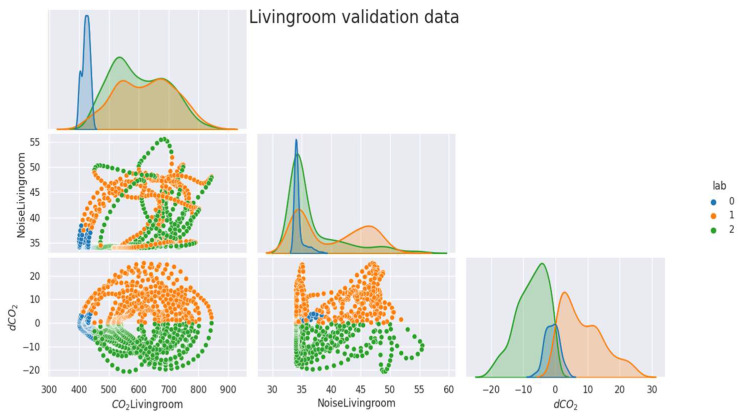
Distribution of data by class for the living room’s validation data.

**Figure 7:**
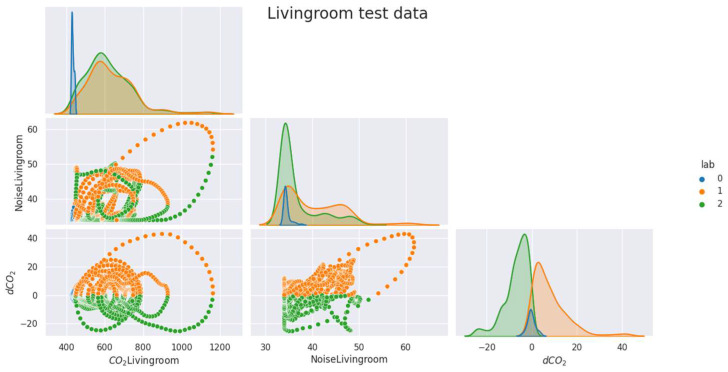
Distribution of data by class for the living room’s test data.

**Figure 8:**
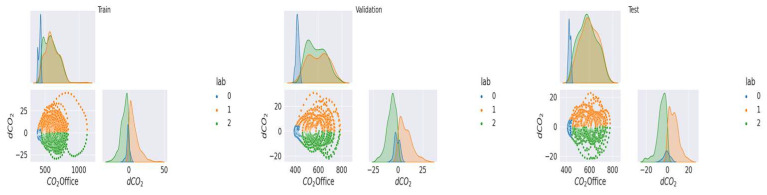
Distribution of data by class for the office.

**Figure 9:**
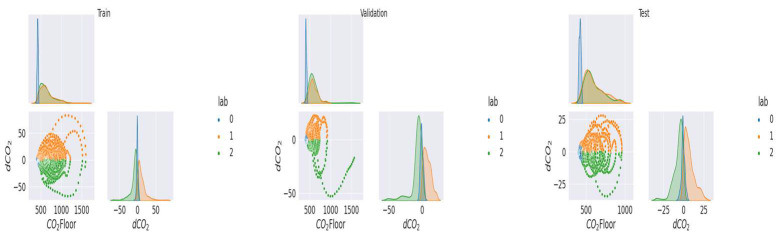
Distribution of data by class for the floor.

As mentioned above (Section 2.2.1), the data were labeled and separated into training, validation, and test samples. In the Figure 5, Figure 6, Figure 7, Figure 8 and Figure 9, we can observe the distributions of the different variables (CO_2_ and its derivative and noise) by class for each sample (learning, validation, and test). The Figure 5, Figure 6 and Figure 7 represent the training, validation, and test data respectively for the living room, the Figure 8 and Figure 9 show the same but for the office and upstairs. We can notice that the distributions of the training, validation, and test samples are relatively similar, or at least there are no significant differences that could indicate a possible lack of generalization. The criteria defined previously through the expressions (Equations (7)–(9)) will be evaluated on these three samples for each of the rooms (living room, floor, and office). To do this we will compare the states predicted by our model to the states obtained by labeling (Section 2.2.1).

#### 2.2.5. Materials

Our experiments were carried out with a DELL brand laptop from Montpellier in France with an Intel Core i.7 CPU architecture and equipped with an nVidia GM108M (GeForce 930MX) graphics card. We used the Miro (Amsterdam/The Netherlands) collaborative platform to design the diagrams. The implementation codes were written in Python (version 3.10.6), and the neural networks were implemented using the tensorflow library (2.9.2). The Table 2 summarizes the calculation times as well as the parameters trained following two scenarios studied in this work, namely prediction over 30 min and prediction over 1 h.

## 3. Results and Discussion

In this section, we give the results obtained in our work and we analyze these results. As a reminder, in this work we have used logical rules, which are based on the impact of occupancy on the dynamics of the variables used (CO_2_ concentration and noise), to associate occupancy states with our data. We then use a recurrent neural network architecture based on LSTM cells to predict future occupancy states from historical data (Section 2.2).

### 3.1. Description of Data

**Figure 10:**
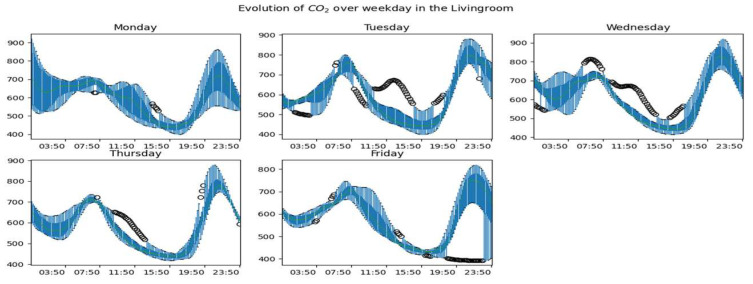
Overall CO_2_ dynamics on weekdays in the living room.

**Figure 11:**
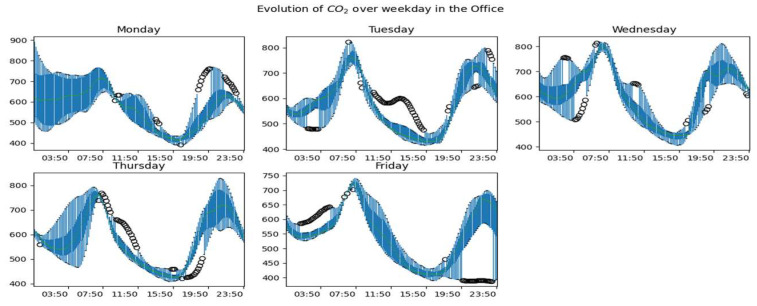
Overall CO_2_ dynamics on weekdays in the office.

**Figure 12:**
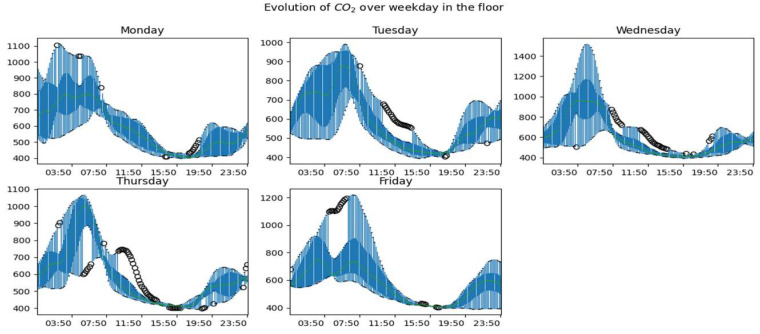
Global CO_2_ dynamics on weekdays in the upstairs.

Figure 10, Figure 11 and Figure 12 show the global dynamics of evolution during working days of the CO_2_ concentration in the living room, the office, and the upstairs, respectively. To obtain these figures, we grouped the data by day and evaluated for each instant the boxplot of data collected at this instant. At the level of each room, we can observe a pattern of occupation taking shape according to the rise and fall of the concentration of CO_2_. We also observe days with greater variability, especially on Mondays, a marked variability, especially between midnight and 6:00. but also in the evening around 22:00. We also notice that the dynamics of CO_2_ on Fridays are more stable than on other days. The CO_2_ upstairs (Figure 12) shows much more variability than in other rooms. Besides these observations, we also have days with atypical CO_2_ dynamics.

**Figure 13:**
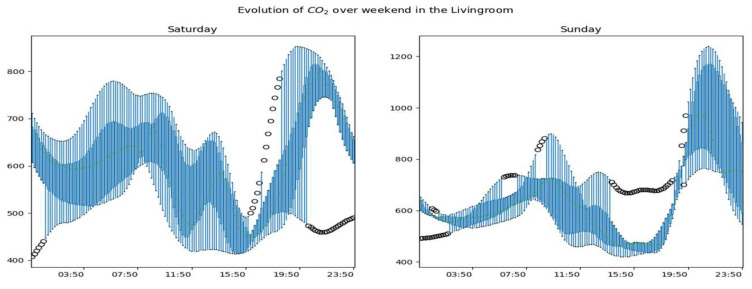
Global dynamics of CO_2_ on weekends in the living room.

**Figure 14:**
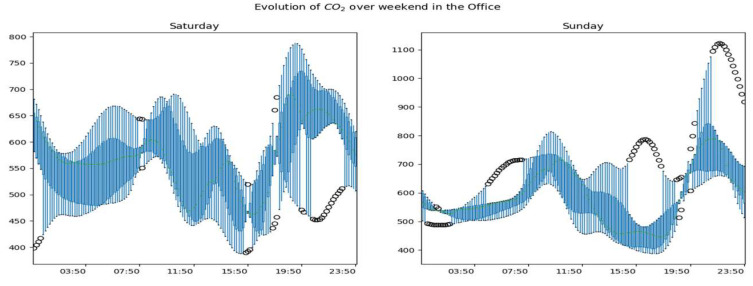
Global CO_2_ dynamics on weekends in the office.

**Figure 15:**
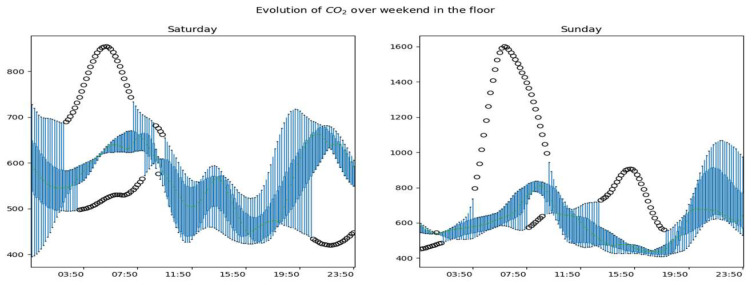
Global CO_2_ dynamics on weekends upstairs.

The Figure 13, Figure 14 and Figure 15 show the overall evolution of CO_2_ in the different rooms on weekends. We observe much more variability in working days than non-working days. We also notice higher data amplitudes on Sundays.

Generally speaking, we notice atypical days whose developments are represented in the figures by black dots. These are days with periodic or global dynamics that are clearly different from other days. We also observe that for a given room, the global dynamics of CO_2_ are substantially similar for working days with obviously more or less accentuated variability by period of the day and/or by day. During non-working days, CO_2_ dynamics are more variable and the highest concentrations are observed on these days.

### 3.2. Labeling

**Figure 16:**
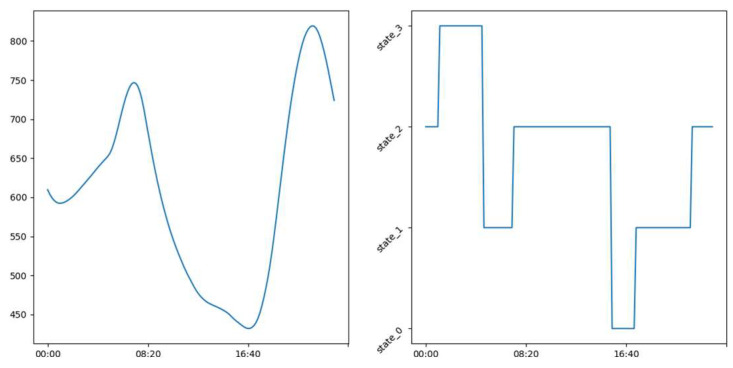
Example showing the occupation schedule after labeling.

**Figure 17:**
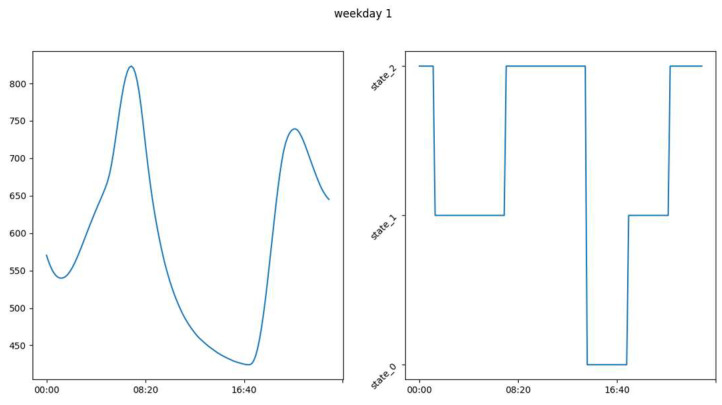
Example of office occupation schedule after labeling.

**Figure 18:**
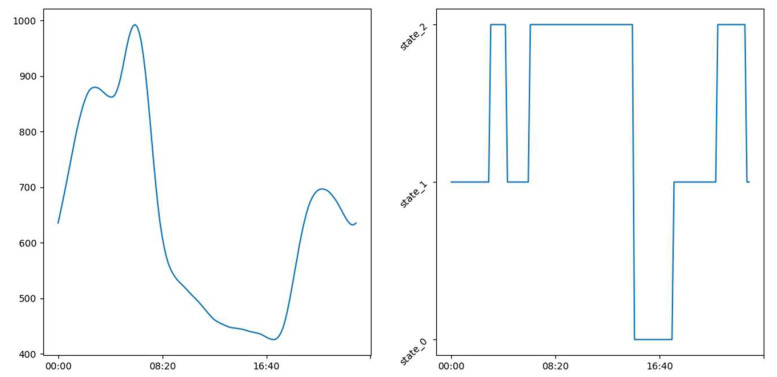
Example of floor occupancy schedule after labeling.

In the Figure 16, Figure 17 and Figure 18 we see examples of the occupation schedule for our different rooms (living room, office, and upstairs). Note that the one-day occupancy schedule represents a succession of different occupancy states on the said day. The schedules are carried over to a full day from 00:00 to 23:50. These schedules are examples and may vary slightly or significantly from day to day. Indeed, the schedules reflect the evolution of CO_2_ in the different rooms (living room, office, and floor) which varies as we saw in Section 3.1. Table 3 shows the distribution of the different occupancy states. This distribution was obtained using the rules in Section 2.2.1. and the thresholds reported in Table 4. As a reminder, our four occupancy states represent, as indicated in Section 2.2.1., the long absence—presence—absence—inactive presence (without noise) for the living room. As a remark, we can say that our different rooms are unoccupied most of the time. We can also notice the relatively low proportions of states (0 and 3) in the living room, which are less than 8% and 12% of the learning sample, respectively. The same observation is made in the office where state 0 represents less than 10% of the learning sample.

From our labeling as illustrated in Figure 16, Figure 17 and Figure 18, we constructed interpretable occupancy schedules with some consistency. These schedules indicate occupation of the building from 0 h until approximately 8 h and from approximately 18:00 until 23:50. The building is unoccupied between 8:00 and 18:00.

### 3.3. Prediction of Occupancy States

Our LSTM network was adjusted during the learning phase to make predictions of the occupancy state of all rooms in the building in a precise instant after 30 min and 1 h. The results are reported in the Table 5 and Table 6 below.

We observe significantly better overall performance for the 30 min horizon; however, the 1 h predictions remain very correct. The performance gap is particularly large for the floor, no doubt due to the variability of the data in this room. We observe better performance upstairs than in the living room for predictions of 30 min; it is the opposite that we observe for predictions after 1 h in the future. Performance in the office remains better in both scenarios.

#### 3.3.1. Prediction in the Living Room

**Figure 19:**
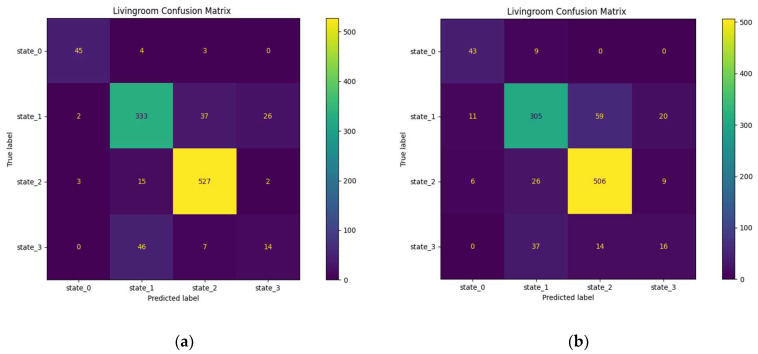
Confusion matrices of the living room: (**a**) lobby confusion matrix for the 30 min horizon; (**b**) lobby confusion matrix for the 1 h horizon.

We have four living room occupancy states, the fourth state is obtained with the addition of the noise variable (Section 2.2.1). In the living room, we observe low proportions for states 0 and 3. In Figure 19a,b, we observe the living room confusion matrices for the 30 min and 1 h horizons.

State (2) is better predicted than the others with a correct prediction ratio of 87–92%. State (0) is 71–90% correctly predicted. The performances for state (3) are very mixed, however, between 44 and 62% of the bad predictions concerning this state are attributed to state (1) which is not a critical error because these two states represent a presence. State (1) is 81–83.6% correctly predicted with 9.8–11.5% of the errors attributed to state (3). This high proportion of state (3) prediction errors is attributable to several factors: the low representativeness of this state in our training sample (Table 3) and/or the relevance of the rules for assigning this state.

#### 3.3.2. Prediction in the Office

**Figure 20:**
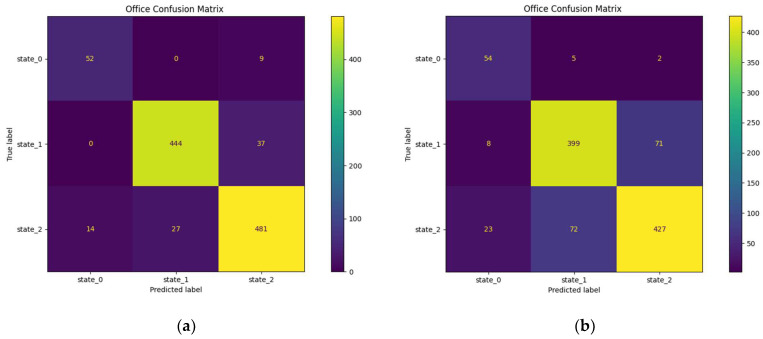
Confusion matrices of the office: (**a**) office confusion matrix for the 30 min horizon; (**b**) office confusion matrix for the 1 h horizon.

In the office, the overall prediction performance is significantly better than in the other rooms, as shown in Figure 20a,b.

The prediction performance of the three office occupancy states is between 63.5%, which represents the proportion of correct prediction of state (0) in the 1 h prediction scenario, and 91.2%, which represents the proportion of correct prediction of state (2) in the 30 min prediction scenario. Note that 27% of the prediction errors of state (0) in the first scenario are attributed to state (2).

#### 3.3.3. Prediction in the Upstairs

**Figure 21:**
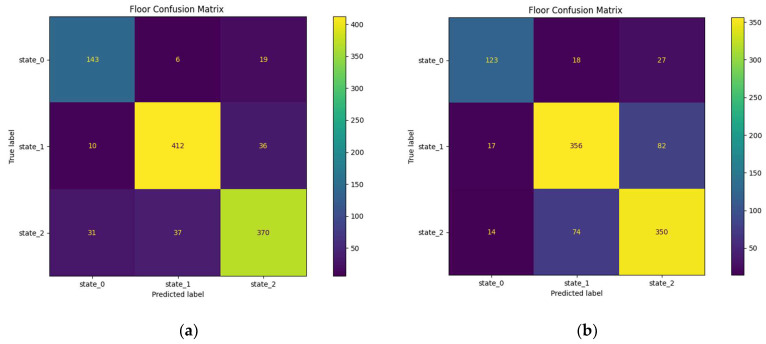
Confusion matrices of the upstairs: (**a**) upstairs confusion matrix for the 30 min horizon; (**b**) upstairs confusion matrix for the 1 h horizon.

Unlike the previous rooms where the best-predicted state is state (2), here it is state (1) which has the best prediction score between 79.4 and 90.5% as you can see from the confusion matrix below (Figure 21a,b). It is also the part with the highest proportion of state (0) (Table 3) predicted at 77.7–79.9% with 9–16.8% of errors attributed to state (2).

#### 3.3.4. Results without the State (3)

In this section we expose the results obtained by removing the state (3), the methodology described in Section 2.2 remains identical. Through the results reported in Table 7 below, we see a very clear improvement in prediction performance in the living room and a slight improvement in performance in the other two rooms. This confirms the sharing of information between rooms within the network (Figure 4) and informs us of the irrelevance of this state (3).

## 4. Conclusions

In this work, we presented a strategy for predicting the occupancy state of a building on the horizons of 30 min and 1 h using in-advance labeling and LSTM networks on environmental data (CO_2_ and noise). We used an LSTM architecture with three parallel inputs allowing us to exploit the information of each room of the building and to return the state of occupation of the building at a given moment in the future. At the end of this work, several observations stand out on a number of points such as:

- The model we have developed links data from several rooms in the same building to provide a prediction of the occupancy status of all rooms in the building at a specific time after 30 min and 1 h in the future.
- The overall prediction performance varies between 78 and 92% (Table 5 and Table 7).
- High prediction errors for state (3) are due to its low representativeness. This is not the most relevant, and can be removed from the prediction because state (3) is included in state (1). However, for cases where the presence without noise (state (3)) represents a non-negligible proportion of the occupancy states, it may be interesting to keep it.
- Removing state (3) significantly improves prediction performance in the living room and slightly in the other two rooms.
- Due to the lack of label data, we used manual labeling to separate training data into classes, which caused issues with the unbalanced representativeness of the states in the dataset. Future works may involve bigger datasets with more information related to the states which we can use to improve training.

The results we obtained in this work are very encouraging and we plan for future work to exploit a larger amount of data. Thus, we will study different labeling strategies (clustering) with possibly the use of resampling algorithms to balance the representativeness of the states.

## Figures and Tables

**Table 1:** Summary of sensor data.

	Count	Mean	Std	Min	25%	50%	75%	Max
CO_2_ living room (ppm)	10,713	607.58	127.13	392.56	510.98	598.72	689.42	1239.41
Noise living room (dB)	10,713	37.73	5.35	34.0	34.0	34.67	40.73	65.69
CO_2_ floor (ppm)	10,713	591.34	171.63	390.67	462.70	554.26	661.22	1601.94
CO_2_ office (pm)	10,713	581.12	105.53	387.08	497.47	576.38	654.15	1122.76

**Table 2:** Calculation time and number of parameters trained.

Prediction Time	3 States		4 States	
	N Param	Process Time (s)	N Param	Process Time (s)
30 min	343	298.57	354	286.12
1 h		288.69		283.59

**Table 3:** Breakdown of states.

	Living Room			Office			Floor		
	Train	Valid	Test	Train	Valid	Test	Train	Valid	Test
state_0	241	119	53	308	129	61	644	197	168
state_1	1017	360	395	1414	423	478	1252	383	455
state_2	1573	507	546	1486	513	522	1312	485	438
state_3	377	79	67	nan	nan	nan	nan	nan	nan

**Table 4:** Labeling thresholds.

	CO_2_	dCO_2_	Noise
state_0	≤445	nan	nan
state_1	>445	>0	>34
state_2	>445	<0	≤34
state_3	>445	>0	≤34

**Table 5:** Performance of the model on the prediction of the occupancy state over 30 min.

	Test			Validation			Training		
	Living Room	Floor	Office	Living Room	Floor	Office	Living Room	Floor	Office
Precision	85%	87.1%	91.9%	87.4%	91.6%	93.6%	86.2%	89.4%	90%
Recall	86.4%	86.9%	91.8%	87.9%	91.3%	93.5%	86.5%	89.3%	89.9%
F1-score	85.6%	87%	91.9%	87.5%	91.3%	93.5%	86.3%	89.3%	89.9%

**Table 6:** Performance of the model on the prediction of the occupancy state over 1 h.

	Test			Validation			Training		
	Living Room	Floor	Office	Living Room	Floor	Office	Living Room	Floor	Office
Precision	80.9%	78.2%	83.4%	78.6%	84.8%	87.4%	78.1%	83.7%	84.2%
Recall	82%	78.1%	82.9%	80.8%	84.1%	87.4%	79.5%	83.7%	83.8%
F1-score	81.3%	78.1%	83%	79%	84.3%	87.4%	77.6%	83.7%	83.9%

**Table 7:** Model performance on occupancy state prediction for occupancy scenarios without state (3).

	Test						Validation						Training					
	Living Room		Floor		Office		Living Room		Floor		Office		Living Room		Floor		Office	
	1/2 h	1 h	1/2 h	1 h	1/2 h	1 h	1/2 h	1 h	1/2 h	1 h	1/2 h	1 h	1/2 h	1 h	1/2 h	1 h	1/2 h	1 h
Precision	92%	89.1%	87.7%	79%	93.6%	85.6%	93.8%	87%	90.9%	82.7%	93.1%	86.5%	89.9%	89%	89.7%	86.4%	93.6%	86.1%
Recall	92%	89.1%	87.7%	78.9%	93.5%	85.4%	93.7%	87%	90.8%	82.3%	93%	86.5%	89.5%	89%	89.7%	86.4%	93.6%	86%
F1-score	92%	89%	87.5%	78.9%	93.5%	85.4%	93.7%	87%	90.8%	82.4%	93%	86.5%	89.7%	89%	89.7%	86.4%	93.6%	86%

## Data Availability

Data are contained within the article.
